# Preimplantation genetic diagnosis of hemophilia A

**DOI:** 10.1186/s12959-016-0098-9

**Published:** 2016-10-04

**Authors:** Ming Chen, Shun-Ping Chang, Gwo-Chin Ma, Wen-Hsian Lin, Hsin-Fu Chen, Shee-Uan Chen, Horng-Der Tsai, Feng-Po Tsai, Ming-Ching Shen

**Affiliations:** 1 Department of Genomic Medicine and Center for Medical Genetics, Changhua Christian Hospital, Changhua, Taiwan; 2 Department of Genomic Science and Technology, Changhua Christian Hospital Healthcare System, Changhua, Taiwan; 3 Department of Obstetrics and Gynecology, College of Medicine and Hospital, National Taiwan University, Taipei, Taiwan; 4 Department of Medical Genetics, National Taiwan University Hospital, Taipei, Taiwan; 5 Department of Life Science, Tunghai University, Taichung, Taiwan; 6 Department of Obstetrics and Gynecology, Changhua Christian Hospital, Changhua, Taiwan; 7 Institute of Biochemistry, Microbiology and Immunology, Chung Shan Medical University, Taichung, Taiwan; 8 Department of Medical Laboratory Science and Biotechnology, Central Taiwan University of Science and Technology, Taichung, Taiwan; 9 Po-Yuan Women’s Clinic and IVF Center, Changhua, Taiwan; 10 Department of Internal Medicine, and Thrombosis and Hemostasis Center, Changhua Christian Hospital, Changhua, Taiwan

**Keywords:** PGD, ARMS-qPCR, Linkage analysis, STR marker, Polymorphism

## Abstract

Preimplantation genetic diagnosis (PGD) is a powerful tool to tackle the transmission of monogenic inherited disorders in families carrying the diseases from generation to generation. It currently remains a challenging task, despite PGD having been developed over 25 years ago. The major difficulty is it does not have an easy and general formula for all mutations. Different gene locus needs individualized, customized design to make the diagnosis accurate enough to be applied on PGD, in which the quantity of DNA is scanty, whereas timely laboratory diagnosis is mandatory if fresh embryo transfer is desired occasionally. Indicators for outcome assessment of a successful PGD program include the successful diagnosis rate on blastomeres (Day 3 cleavage-stage embryo biopsy) or trophectoderm cells (Day 5/6 blastocyst biopsy), the implantation rate per embryo transferred, and the livebirth rate per oocyte retrieval cycle. Hemophilia A (HA) is an X-linked recessive bleeding disorder caused by various types of pathological defects in the factor VIII gene (*F8*). The mutation spectrum of the *F8* is complex, according to our previous report, including large segmental intra-gene inversions, large segmental deletions spanning a few exons, point mutations, and total deletion caused by chromosomal structural rearrangements. In this review, the molecular methodologies used to tackle different mutants of the *F8* in the PGD of HA are to be explained, and the experiences of successful use of amplification refractory mutation system-quantitative polymerase chain reaction (ARMS-qPCR) and linkage analysis for PGD of HA in our laboratory are also provided.

## Background

Preimplantation genetic diagnosis (PGD) had become a standard of care when dealing with stopping the transmission of the heritable disease from generation to generation since it was firstly introduced in 1990 [1, 2]. The gold standard of molecular technology used for PGD nowadays is the coamplification of the polymorphic microsatellite linkage markers [3, 4]. However, such techniques cannot avoid the possibility of recombination occurred within the segment which separated the linked polymorphic markers and the disease loci, and it is advised to combine more informative linkage markers to reduce the chance of misdiagnosis. On the other hand, direct mutation detection assay, either rapid PCR-based or the more time-consuming sequencing-based genotyping platforms, is prone to allele dropout (ADO), which may ensue a catastrophic false-negative misdiagnosis in PGD of autosomal dominant monogenic disorder [4, 5].

Hemophilia A (HA) (OMIM 306700), a bleeding disorder which causes long-term disability, is a X-linked recessive disorder and its causative gene is situated at Xq28, the factor VIII (*F8*) gene, is a serious threat for public health in Taiwan, and we had first published its mutation spectrum in the Taiwanese population in 2008 [6]. The mutation spectrum included rearrangements such as intron 1 inversions (INV1) and intron 22 inversions (INV22), large deletions spanning for consecutive exons, small deletions involving only a few base pairs, and point mutations [6]. The broad spectra of F8 mutations have also been reported in several other studies [7–9]. The genotyping itself for the *F8* is already a daunting task given its complicated existing mutations patterns, let alone the PGD. In spite of the challenges, we managed to tackle these difficulties in a few families who came to our hospital seeking for PGD. Meanwhile, a few similar efforts had been reported from other laboratories [10, 11], which indicates PGD for HA is feasible, at least in those families the mutation has been confirmed. Here we will give a concise review of PGD for HA, including the different molecular technologies used to tackle different mutation patterns, and also to cite a few of our experience with successful outcome, that is, to give birth to normal unaffected babies in families suffered from HA.

## Review

### Mutation spectrum of the F8 gene, genotyping strategies, and possible PGD approaches

Since the publication of the sequence of the *F8* in 1984, more than 2000 gene mutations causing HA have been described and these are catalogued in the Human Gene Mutation Database (HGMD; http://www.hgmd.cf.ac.uk/ac/index.php) and Factor VIII Variant Database (http://www.factorviii-db.org/). In 2008, we had first published the mutation spectrum in the Taiwanese population [6]. Of 31 unrelated HA patients (19 severe and 10 moderate/mild males, and 2 severe females), 12 (38.7 %) and 1 (3.2 %) severe males were genotyped with INV22 and INV1 respectively. The *F8* defects in the remaining 18 inversion-negative patients cover a wide spectrum, in which 17 different mutations were identified (10 missense and 3 nonsense mutations, and 2 small and 2 large deletions). Eleven of these mutations are novel and unique, confirming a high diversity of molecular defects in HA [6]. A systematic review for data from 30 studies on 5383 patients had been reported and showed 45 % of HA had INV22, 2 % INV1, 3 % large deletions, 16 % small deletions or insertions, and 28 % point mutations (15 % missense mutations, 10 % nonsense mutations, and 3 % splicing site mutations). In 4.6 % of patients, the mutation was unknown [12]. Overall, with the exceptions of recurrent INV22 and INV1, no mutation hot spots have been identified.

**Fig. 1:**
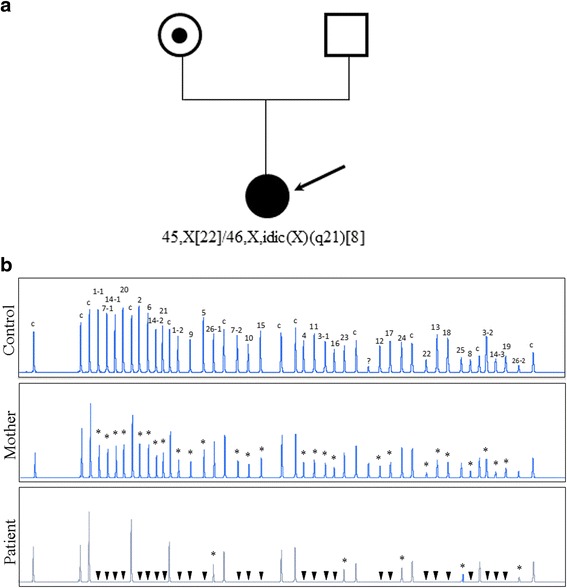
Genetic testing for a female patient (indicated by an arrow) with severe hemophilia A. **a** Cytogenetic analysis identifies a 45,X [22]/46,X,idic(X)(q21) [8] mosaicism, indicating at least one *F8* allele loss. **b** MLPA analysis for the *F8* gene of the patient detects only copy of exon 23–26 peaks indicating an exon 1–22 deletion in the allele on her “morphologically-normal” X-chromosome. MLPA for the patient’s mother detects about 1/2 DNA dosage of exon1-22 indicating a carrier of exon 1–22 deletion. Arabic numbers, the exon numbers of the *F8* gene. “c”, the internal controls used in MLPA. “?”, an unexpectedly amplified peck which is not illustrated in the instruction of the MLPA FVIII kit, SALSA P178. “*”, loss of one copy in exons. “▼”, loss of two copies in exons

There are a number of different approaches for the genotyping of HA (Table 1). For reasons of rapid and smart screening, however, targeted mutation analysis for the recurrent INV22 and INV1 has become the first test assessed in patients (particularly in severely affected hemophiliacs). INV22 can be detected by Southern blotting or, more time- and labor-saving choice, by long-distance polymerase chain reaction (long-distance PCR) or inverse PCR (I-PCR) [13, 14]. INV1 is typically detected by multiplex PCR [15]. Other mutations responsible for HA are mostly point mutation and small deletion/insertion in the *F8* gene and their spectrum is quite complex. In these cases, mutation can be detected by PCR with a number of screening methods (e.g., single strand conformational polymorphism, conformation sensitive gel electrophoresis, amplification and mismatch detection, denaturing gradient gel electrophoresis) followed by direct DNA sequencing [16–21]. For female patients with only one mutation detected and also in those females suspected to be carriers but no mutation could be found, gene dosage assays such as multiplex ligation-dependent probe amplification (MLPA) should be applied to screen for the underlying exon deletions since deletions in single allele usually escape detection by the PCR-based analysis, due to the masking of the non-deleted allele. In Fig. 1, we exemplified the MLPA finding of a female HA patient who was karyotyped as 45,X [22]/46,X,idic(X)(q21) [8] mosaicism. Her aberrant X-chromosome (idic(X)(q21)) do not contain the Xq22q28 (and thus *F8* gene) and familiar follow-up studies demonstrate this anomaly is of *de novo*. PCR amplification for exon1-22 of the *F8* is failure in patient but is successful in her parents. Through the MLPA analyses, it is evidenced that the patient carries an exon 1–22 deletion in the allele on her “morphologically-normal” X-chromosome, which is inherited from her mother (Fig. 1).

**Table 1:** Genotype-phenotype relationship, genetic testing and preimplantation genetic diagnosis (PGD) in hemophilia A

Mutation type	Frequency of occurrance^a^	Clinical severity^b^	Test method	PGD method
Inversion • INV22 • INV1	47 % • 45 % • 2 %	Severe	• I-PCR (for INV22) • Long-distance PCR (for INV22) • Southern blotting (for INV22) • Multiplex PCR (for INV1)	• Linkage analysis
Point mutation • Missense • Nonsense • Splicing site	28 % • 15 % • 10 % • 3 %	Mild, Moderate, Severe • Mild, Moderate (majority) • Severe (majority) • Severe (majority)	Direct DNA sequencing	• ARMS-qPCR • Linkage analysis
Small deletion/insertion (<1 exon)	16 %	Severe (majority)	Direct DNA sequencing	• ARMS-qPCR • Linkage analysis
Large deletion (≥1 exon)	3 %	Severe (majority)	MLPA	• Linkage analysis
Others (e.g., Complex rearrangement)	NA	Severe (majority)	Depending on mutation entities	• Linkage analysis

*MLPA* multiplex ligation-dependent probe amplification, *I-PCR* inverse polymerase chain reaction, *ARMS* amplification refractory mutation system, *NA* not available

^a^See the review in Gouw et al., [12]

^b^HA patients are clinically divided into three different severities based on the residual FVIII coagulant activity (FVIII:C): severe (FVIII:C < 1 % of normal level), moderate (FVIII:C is 1–5 % of normal level) and mild (FVIII:C is 5–30 % of normal level)

Given the marked morbidity associated with severe HA, PGD has become a feasible option for couples at risk of having a child with HA since it reduces the risk of termination of affected pregnancies. Gender selection by fluorescence in situ hybridization (FISH) and transfer of only female embryos is a simple strategy for X-linked recessive disorders, such as HA, and has been adopted in many clinics [11]. However, in practice, gender selection is illegal in some countries (e.g., Taiwan) and methods allowing the correct and more definitive diagnosis of the HA status of every embryo are more desirable because the number of embryos available for transfer is increased. PGD involving whole genome amplification (WGA) step was broadly applied to mutation detection strategies, but the high rate of amplification bias renders WGA an imperfect option [22]. Recently, co-amplification of polymorphic microsatellite markers, linked with the targeted mutation, had been the gold-standard genotyping strategy for PGD [4, 23–26] (Table 1). A linkage approach using polymorphic markers located near the mutation allows monitoring the occurrence of allele dropout, a known problem associated with PCR amplification bias in PGD. Below, we describe our experience with two HA families seeking for PGD.

### Experience of PGD of hemophilia A in our laboratory

**Fig. 2:**
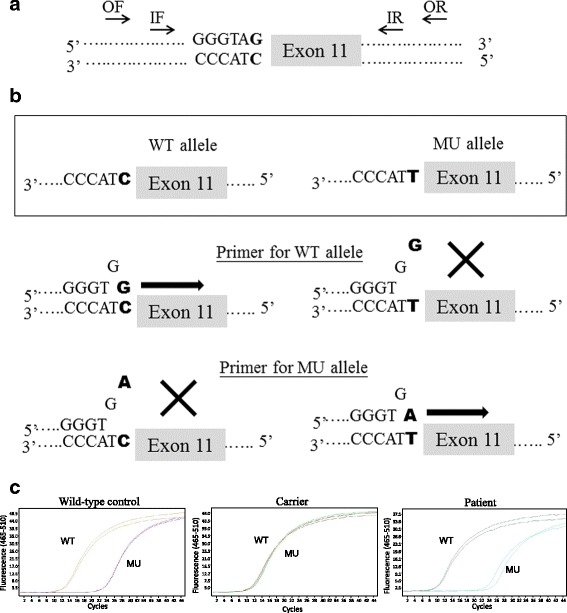
Schematic diagram of a duplex-nested ARMS-qPCR for PGD of a splicing-site point mutation, located at the junction of intron 10 and exon 11 of *F8*, c.1538-1G > A (bold letter). **a** Primers for duplex-nested PCR were first designed to amplify the region covering the position of the mutation. OF and OR indicate the outer primer set, and IF and IR indicate inner primer set. **b** Primers specific for the amplification of the wild-type (WT) and mutant (MU) alleles, respectively, were subsequently used for ARMS-qPCR where the duplex-nested PCR amplicon was used as DNA template. **c** Representative ARMS-qPCR results for wild-type control (homozygous WT/WT or hemizygous WT), female carrier (heterozygous WT/MU), and affected male individual (hemizygous MU)

**Fig. 3:**
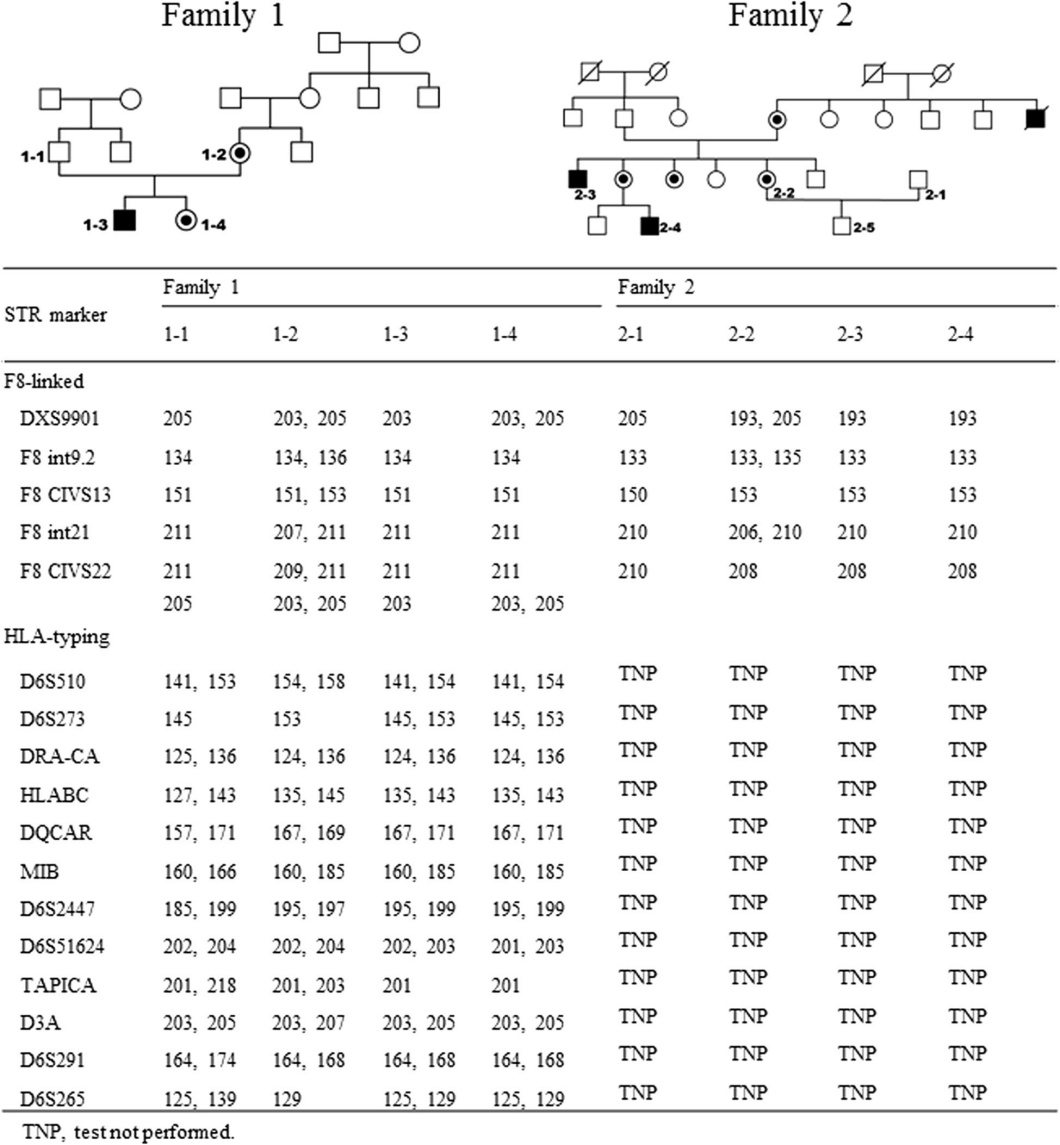
Exemplified PGD of *F8* defects for two hemophilia A families: family 1 (c.1538-1G > A mutation) and family 2 (INV22). PGD was performed using ARMS-qPCR, together with linkage analysis for five informative short tandem repeat (STR) markers ordered from centromere (*top*) to telomere (*bottom*). The numbers in STR markers represent the sizes of PCR amplicons in base pair (bp). In family 1, human leukocyte antigen (HLA) typing with 12 STR markers was also performed. PGD for hemophilia A resulted in a birth of healthy girl (1–4), who was HLA matched to the affected sibling (1–3). In family 2, PGD for INV22 was directly performed by linkage analysis. The maternal allele linked to INV22 was evidenced by comparing the STR profile with that of case 2–3 and 2–4. In the pedigree, squares represent males, and circles represent females. Line through, filled, dotted and open the symbols represent deceased, affected, carrier and unaffected individuals respectively

Two Taiwanese couples were referred to our center for PGD of HA. Preliminary genetic testing of *F8* for two couples showed the two wives are both HA carriers: one has a splicing site mutation, c.1538-1G > A, and the other has a common INV22. For the family with c.1538-1G > A mutation, an in-house developed duplex-nested ARMS-qPCR was customized designed for PGD [4, 5]. The optimized PGD protocol was then performed to detect the disease-causing mutation in embryos acquired after ovarian stimulation. Seven single blastomeres biopsied from corresponding day-3 (8-cell cleavage-stage) embryos were collected and examined independently in a sterile PCR tube. Each blastomere cell was lysed with 125 μg/mL proteinase K at 50 °C for 60 min and inactivated at 99 °C for 4 min. Duplex-nested PCR was then used to amplify the intron 10–11 region of *F8* gene, which includes the targeted mutation. The two primer sets, designed based on the reverse strand’s *F8* gene sequence, were OF: CTGAGGACCCATTACCCTGA and OR: CCTGCAACAGTGCTACATGC for the first PCR (amplicon size: 937 bp), and IF: CTTGCTCCCTTTCCTCACAG and IR: TGGGGAGGATCAGCTAGAGA for the secondary PCR (amplicon size: 757 bp) (Fig. 2a). The first PCR was carried out in a 40 μL reaction, consisting of 1X PCR buffer, 1.25 mmol/L MgCl_2_, 0.35 mmol/L dNTP, each 0.5 μmol/L of OF and OR primer, 1X GC-RICH solution, and 1U Faststart Taq DNA polymerase (Roche Diagnostics GmbH, Mannheim, Germany). The cycling conditions were 95 °C, 5 min, followed by 25 cycles of 95 °C, 30 s, 55 °C, 30 s and 71 °C, 1 min, and a final extension at 71 °C, 2 min. The PCR products were directly subjected to the second round of PCR by adding 10 μL PCR supplement with a similar PCR mixture to that used in the first PCR, except for the primer set (IF and IR). Cycling conditions were similar to the first PCR, but the number of cycles in the second step was increased from 25 to 40. To ensure an accuracy of the PCR, amplified fragments were confirmed by direct sequencing. For ARMS-qPCR, two sequence-specific forward primers, modified with a mismatch at the penultimate nucleotide position of the mutation site to increase the specificity of the reaction, were designed: (MUF: TATGGTTTTGCTTGTGGGTGA for the mutant allele and WTF: TATGGTTTTGCTTGTGGGTGG for the wild-type allele). The two forward primers were respectively paired with the same reverse primer 3rdR: TGAGGAGAGGGCCAATGAGT (amplicon size:198 bp) (Fig. 2b). The ARMS-qPCR performed on the LightCycler 480 Real-Time PCR System (Roche, Mannheim, Germany) in a 20 μL reaction, consisted of 0.5 ng of the duplex-nested PCR product, 0.5 μmol/L of each primer, 1X SYBR Green PCR Master Mix (Finnzymes, Espoo, Finland). Cycling conditions were: 95 °C, 10 min, followed by 45 cycles at 95 °C, 10 s, 60 °C and 60 s. The exemplified results of ARMS-qPCR were shown in Fig. 2c. Of the seven blastomeres examined, two were reported as affected embryos with the homo- or hemizygous c.1538-1G > A mutation, one was known as a heterozygous carrier, and the remaining four were presented as same as wild-type pattern without the mutation. However, only one unaffected embryo kept good morphology and was transferred on day 5. After 39 weeks of uneventful gestation, one healthy baby girl was born successfully with a birth weight of 3040 g. Postnatal genotyping confirmed the girl to be an unaffected carrier and, interestingly, she was HLA-compatible with her older brother affected by HA (see family 1 in Fig. 3).

The INV22 of *F8* is one of the most frequent cause of severe HA, known as a result of homologous recombination between the int22h-1 region within the *F8* locus and either int22h-2 (Inv22 type II) or int22h-3 (Inv22 type I), which lie nearly 400 kb distal to *F8* [27]. The gene rearrangements tend to increase the difficulty of PGD experimental design performed in the affected families. In the second PGD family with *F8* INV22 mutation, the couple (2–1 and 2–2) has had a healthy boy and they would like to get more babies without HA. We performed 3 PGD cycles of HA during the period of Sep. 2014 to Dec. 2015 using short tandem repeat (STR) markers and capillary electrophoresis for direct linkage analysis. Five informative STR markers distributed within or near the flanking region of the *F8* were selected for PGD performing (see family 2 in Fig. 3). Among a total of 19 examined embryos, 5 wild types and 4 INV22 carriers were selected and transferred during the period. Unfortunately, pregnancy outcome did not occur as expected, possibly due to ad maternal age, embryo morphology, development and other abnormalities. Despite the fact that no pregnancy was achieved in the PGD experience so far, they are still willing to keep trying.

## Discussion

The outcome indicators of PGD can be classified as successful diagnosis rate (the number of embryos which diagnosis was made/the total number of embryos being biopsied), implantation rate (the number of embryos implanted/the total number of embryos being transferred), and the live-birth rate (the rate of liveborn pregnancy per transferred cycle or the rate of liveborn pregnancy per oocyte-retrieval). It is now still under debate whether frozen or fresh embryo transfer can achieve a better outcome against the other. However, it is vitally important that PGD laboratories developed a timely genotyping platform to cope with the need of fresh embryo transfer, especially when Day 5/6 blastocyst biopsy is undertaken. For rapid PGD of HA, the direct mutation detection, e.g., ARMS-qPCR, can greatly increase the reliability of mutation detection in embryos with small insertions/deletion and point mutations (see the exemplified couple 1). However, for large and complex *F8* defects, e.g., INV1 and INV22, PGD by direct genotyping is not easily feasible and indirect linkage analysis with informative markers may be considered (see the exemplified couple 2). Of noted, the chance of recombination between the markers and mutation can lead to small diagnostic error and some families may not be informative for any of the available markers.

Recently, it is noteworthy that because of the popularity of preimplantation genetic screening (PGS), there is a growing need of concurrent PGD/PGS. At the moment the strategies used in PGS, if we exclude the outdated FISH-based diagnostics [28], include array-based (either array comparative genomic hybridization or single nucleotide polymorphism chromosomal microarray) techniques [29–31], q-PCR based techniques [32, 33], and next generation sequencing (NGS)-based techniques [34, 35]. Some of the techniques had been reported to successfully being applied in PGD combined with PGS [36–39]. It is inevitable that in the near future, women will opt for select the unaffected embryos with certain heritable monogenic disorders, such as HA, as well as the euploid embryos which will reduce the chance of abortion due to aneuploidy in the later gestational period or improved the implantation rate as many researchers advocated [40]. However, it will be undisputable only after more convincing randomized trials to prove the efficacy of PGS, the combination of PGD and PGS should be offered to all women underwent PGD [41]. Those couple who opted for PGD combined with PGS should be counseled that double selection will inevitable reduce the number of embryos which are classified as “suitable” for transfer, thereby reducing all the outcome indicators of PGD, the most important live-birth rate is certainly included.

## Conclusions

PGD of HA by direct mutation analysis or indirect linkage analysis has become a feasible option for couples at risk of having an affected child. However, given the broad spectra of the *F8* mutations, genetic counseling along with the technical aspects of the accuracy and limitations of tests should be provided for couples who request PGD.

## Abbreviations

ADO: Allele dropout
ARMS-qPCR: Amplification refractory mutation system-quantitative polymerase chain reaction
FISH: Fluorescence in situ hybridization
HA: Hemophilia A
HGMD: Human gene mutation database
INV1: Intron 1 inversion
INV22: Intron 22 inversion
I-PCR: Inverse polymerase chain reaction
long-distance PCR: Long-distance polymerase chain reaction
MLPA: Multiplex ligation-dependent probe amplification
NGS: Next generation sequencing
PGD: Preimplantation genetic diagnosis
PGS: Preimplantation genetic screening
STR: Short tandem repeat
WGA: Whole genome amplification
